# Systemic Treatment for Advanced and Metastatic Non-Clear Cell Renal Cell Carcinoma: Examining Modern Therapeutic Strategies for a Notoriously Challenging Malignancy

**DOI:** 10.15586/jkcvhl.v10i3.295

**Published:** 2023-09-26

**Authors:** Jake Drobner, Daniella Portal, Karie Runcie, Yuanquan Yang, Eric A. Singer

**Affiliations:** 1 Division of Urology, Rutgers Robert Wood Johnson Medical School, The State University of New Jersey, New Brunswick, NJ, USA;; 2 Division of Hematology/Oncology, New York-Presbyterian/Columbia University Medical Center, New York, NY, USA;; 3 Genitourinary Oncology Section, Division of Medical Oncology, The Ohio State University Comprehensive Cancer Center-James Cancer Hospital, Columbus, OH, USA;; 4 Division of Urologic Oncology, The Ohio State University Comprehensive Cancer Center, Columbus, OH, USA

**Keywords:** chromophobe, immunotherapy, non-clear cell renal cell carcinoma, papillary, tyrosine-kinase inhibitor

## Abstract

Non-clear cell renal cell carcinoma (nccRCC) is a heterogeneous group of malignancies that represents 25% of renal cell carcinoma (RCC) cases. Treatment for non-clear cell histologies is mostly based on evidence from small phase II clinical trials or extrapolated from successful therapies in clear cell RCC because of the low incidence of non-clear cell pathology. Advances in genomic profiling have improved clinicians’ understanding of molecular targets for nccRCC, such as altered mesenchymal epithelial transition (*MET*) gene status and fumarate hydratase (*FH*) gene inactivation, but patient outcomes remain poor and optimal management of this disease remains unclear. This review assesses outcomes by histologic subtype from 27 prospective and 13 ongoing clinical trials to identify therapeutic strategies for advanced or metastatic nccRCC. Vascular endothelial growth factor tyrosine kinase inhibitors (TKI), such as sunitinib, and mammalian target of rapamycin (mTOR) inhibitors, such as everolimus, have demonstrated efficacy and remain viable treatment options, with a preference for sunitinib. However, everolimus is preferred in patients with chromophobe RCC because folliculin (*FLCN*) gene mutations upregulate the mTOR pathway. Novel TKIs, such as cabozantinib, show improved outcomes in patients with papillary RCC because of targeted *MET* inhibition. Platinum-based chemotherapy continues to be the recommended treatment strategy for collecting duct and medullary RCC. Clinically meaningful antitumor activity has been observed across all non-clear cell histologies for immune checkpoint inhibitors, such as nivolumab, pembrolizumab, and ipilimumab. Ongoing trials are evaluating novel tyrosine kinase inhibitor and immunotherapy combination regimens, with an emphasis on the promising *MET*-inhibitor cabozantinib and pembrolizumab plus lenvatinib.

## Introduction

Renal cell carcinoma (RCC) is one of the most common malignancies in the United States. With 79,000 new patients reported by the American Cancer Society in 2022, it is currently the sixth most frequent cancer in men and the ninth most frequent cancer in women (1). RCC is divided into two major histologic classes: clear cell, which represents approximately 75% of RCC, and non-clear cell, which represents the remaining 25% (2). Non-clear cell renal cell carcinoma (nccRCC) is a heterogenous entity that is further divided into several subtypes, such as papillary renal cell carcinoma (pRCC) and chromophobe renal cell carcinoma (chRCC), which together represent about 80% of nccRCC. The remaining 20% of nccRCC is composed of several rarer phenotypes, including collecting duct (CDC), renal medullary (RMC), translocation (tRCC), and unclassified (uRCC) (3).

Sarcomatoid differentiation can occur in both ccRCC and nccRCC at variable proportions. Over the past few decades, advances in molecular profiling have enabled a deeper understanding of the specific genetic and metabolic changes associated with each unique histologic class of RCC. RCC is now known to be associated with at least 17 different genes that dysregulate the ability of tumor cells to respond to changes in oxygen, iron, nutrients, and energy levels (4).

The management of RCC has evolved rapidly with the introduction of drugs that can target these alterations in oxygen metabolism and cellular proliferation (5, 6). Nevertheless, treatment for RCC remains particularly challenging, as most RCCs are clinically silent, and therefore the diagnosis is often not made until the disease is advanced or has metastasized (7). In the early 2000s, novel therapies that focused on the downstream products of the von Hippel-Lindau/hypoxia-inducible factor (VHL/HIF) pathway, such as vascular endothelial growth factor (VEGF), transforming growth factor α (TGFα), and platelet-derived growth factor β (PDGFβ), demonstrated benefit for RCC patients (8, 9). This was clinically significant, given that RCC is considered a chemotherapeutically resistant cancer (with the exclusion of certain nccRCC subtypes) (10).

In the past few years, promising clinical trial data have shifted the standard of care for advanced and metastatic RCC toward immune checkpoint inhibitors (ICIs). However, most randomized phase III trials with data supporting the efficacy of ICIs include only patients with clear cell histology (11, 12), and only up to one-third of patients with nccRCC respond to ICIs based on small phase II studies (13). Furthermore, data for the efficacy of other targeted therapies used in RCC, such as tyrosine kinase inhibitors (TKIs) and inhibitors of mammalian target of rapamycin (mTOR), are limited and must be extrapolated from trials of clear cell histology patients (14, 15). Given that nccRCC represents a distinct pathology, the optimal systemic therapy for patients with nccRCC remains unclear, and therapeutic strategies in nccRCC are currently adapted from the success of various agents in ccRCC treatment. Currently, the National Comprehensive Cancer Network (NCCN) 2022 guidelines for the management of advanced or metastatic nccRCC recommend enrollment in clinical trials or treatment with TKIs, such as cabozantinib or sunitinib (16). The objective of this review is to assess the data from prospective clinical trials on systemic therapies for nccRCC by histologic subtype.

## Methods

A literature search was conducted to identify studies reporting prospective outcomes of systemic therapies in advanced or metastatic nccRCC. The search terms, “non-clear cell renal cell carcinoma,” “advanced non-clear cell renal cell carcinoma,” “metastatic non-clear cell renal cell carcinoma,” “papillary renal cell carcinoma,” “chromophobe renal cell carcinoma,” “translocation renal cell carcinoma,” “collecting duct renal cell carcinoma,” and “renal medullary carcinoma” were used to query the PubMed and Google Scholar databases. All original articles with full texts available reporting prospective clinical trial data between 1981 and July 2023 regardless of the number of patients, the type of therapy, and treatment naivety were included. The main findings were organized by histologic subtype of nccRCC. Publications that contained only retrospective data were excluded. Ultimately, 26 studies and 14 ongoing clinical trials were included for analysis.

## Body: Non-Clear Cell Renal Cell Carcinoma Trials

### Grouped Histology

Grouped histology trials represent prospective clinical trials that excluded ccRCC patients but analyzed outcomes for individual patients with distinct nccRCC subtypes (such as pRCC or chRCC) as an aggregated nccRCC group. The modern therapeutic landscape for nccRCC begins with the development and success of TKIs and mTOR inhibitors for the treatment of clear cell RCC in the early 2000s, which prompted investigators to examine the efficacy of these drugs in non-clear cell histologies (Table 1). In a multicenter phase II trial of 31 nccRCC patients (71% pRCC and 10% chRCC), sunitinib, a VEGF-receptor TKI, demonstrated promising therapeutic activity. Patients taking sunitinib had an objective response rate (ORR) of 35%, a median progression-free survival (mPFS) of 6.4 months, and a 1-year overall survival (OS) of 65% (17). Subsequently, two randomized phase II trials, the European Society of Parenteral and Enteral Nutrition (ESPEN) and American Society of Parenteral and Enteral Nutrition (ASPEN) trials, compared sunitinib with everolimus, an mTOR inhibitor. ESPEN, the first head-to-head comparison of these two drugs, did not demonstrate a statistically significant difference in mPFS (6.1 months for sunitinib vs. 4.1 months for everolimus) or OS (16.2 months for sunitinib vs. 14.9 months for everolimus) across 68 patients (18). ASPEN, which analyzed a larger cohort of patients (n = 108), found that sunitinib significantly increased mPFS when compared to everolimus (8.3 months vs. 5.6 months) but sunitinib did not significantly improve OS, compared to everolimus (19). The Central European Society for Anticancer Drug Research (CESAR) trial, which compared sunitinib (n = 10) to another mTOR inhibitor, temsirolimus (n = 12) reported mPFS of 13.2 months and 9.3 months and an OS of 19.8 months and 19.4 months for sunitinib and temsirolimus, respectively (20). Although the trial was terminated due to low recruitment, and the results were limited due to small sample size and lack of statistical significance, CESAR provided further evidence that sunitinib offers an advantage over mTOR inhibitors, as previously established by both ESPEN and ASPEN.

**Table 1:** Grouped histology.

Agent (trial, year)	Phase	N per group	Histology (%)	Primary endpoint	ORR, % (95% CI)	mPFS, months (95% CI)	OS, months (95% CI)
Sunitinib (2012)^(17)^	II	31	22 (71) papillary 3 (10) chromophobe 5 (16) unclassified 1 (3) Xp11.2 translocation	ORR	35 (19–52)	6.4 (4.2–8.6)	NR, estimated at 25.6 (8.4–42.9) 1-year OS: 65%
Sunitinib vs. everolimus (ESPEN, 2016)^(18)^	II	33 Sunitinib 35 Everolimus	14 (43) papillary 6 (18) chromophobe 4 (12) unclassified 3 (9) translocation unspecified 6 (18) clear cell with sarcomatoid features 13 (38) papillary 6 (17) chromophobe 6 (17) unclassified 4 (11) translocation unspecified 6 (17) clear cell with sarcomatoid features	PFS	9 2.8	6.1 4.1 (P = 0.6)	16.2 14.9 (P = 0.18)
Sunitinib vs. everolimus (ASPEN, 2016)^(19)^	II	51 Sunitinib 57 Everolimus	33 (65) papillary 10 (20) chromophobe 8 (16) unclassified 37 (65) papillary 6 (10) chromophobe 14 (25) unclassified	PFS	18 9	8.3 (8.0–11.4) 5.6 (5.0–5.6) HR: 1.41 (0.03–1.92)	31.5 (14.8–NA) 13.2 (9.7–37.9) HR: 1.12 (0.7–2.1)
Temsirolimus vs. sunitinib (CESAR, 2020)^(20)^	II	12 Temsirolimus 10 Sunitinib	8 (67) papillary 2 (17) chromophobe 1 (8) medullary 1 (8) unclassified 8 (80) papillary 2 (20) unclassified	PFS	NA NA	9.3 13.2 HR: 1.64 (0.65–4.18)	19.4 19.8
Pazopanib (2018)^(22)^	II	29	19 (66) papillary 3 (10) chromophobe 7 (24) unclassified	ORR	28	16.5 (10.9–22.1)	NR
Axitinib (2018)^(23)^	II	40	26 (60) papillary 4 (10) chromophobe 7 (18) MiT translocation 3 (7) others	PFS	37.5	7.4 (5.2–9.5)	12.1 (6.4–17.7)
Everolimus + bevacizumab (2016)^(24)^	II	34	5 (14) papillary 5 (14) chromophobe 2 (6) medullary 23 (66) unclassified	PFS	29	11 (3.8–19.3)	18.5
Everolimus + bevacizumab (2020)^(25)^	II	37	13 (35) papillary 1 (3) translocation unspecified 23 (62) unclassified	PFS	35	13.7 (10.8–16.4)	33.9 (23.3–71.9)
Nivolumab (Checkmate 374, 2020)^(26)^	IIIb/IV	44	24 (55) papillary 7 (16) chromophobe 1 (2) medullary 1 (2) collecting duct 2 (5) translocation unspecified 8 (18) unclassified 1 (2) unreported	High-grade immune-mediated adverse events	13.6 (5.3–27.4)	2.2 (1.8–5.4)	16.3 (9.2–NE)
Atezolizumab + bevacizumab (2020)^(27)^	II	42	12 (35) papillary 10 (29) chromophobe 1 (3) medullary 5 (15) collecting duct 5 (15) *TFE3*translocation 9 (26) unclassified	ORR	26	8.3 months (5.7–10.9)	NR
Cabozantinib + atezolizumab (COSMIC, 2021)^(29)^	Ib	32	15 (47) papillary 9 (28) chromophobe 1 (3) collecting duct 1 (3) fumarate-hydrase deficient 1 (3) MiT-family translocation 1 (3) translocation unspecified 1 (3) poorly differentiated 1 (3) spindle-cell 1 (3) clear cell	ORR	31 (20–44)	9.5 (6.4–18.3) 1-year PFS: 39%	NR
Cabozantinib + nivolumab (2022)^(30)^	II	40	32 (80) papillary 2 (5) translocation unspecified 6 (15) unclassified	ORR	47.5 (31.5–63.9)	12.5 (6.3–16.4)	28 (16.3–NE)
Nivolumab + ipilimumab (Checkmate 920, 2022)^(31)^	IIIb/IV	52	18 (35) papillary 7 (13) chromophobe 1 (2) medullary 2 (4) collecting duct 2 (4) translocation, unspecified 22 (42) unclassified	Grade ≥3 immune-mediated adverse events	19.6 (9.4–33.9)	3.7 (2.7–4.6)	21.2 (16.6–NE)
Pembrolizumab + lenvatinib (KEYNOTE-B61, 2023)^(33)^	II	158	93 (59) papillary 29 (18) chromophobe 6 (4) translocation 9 (6) other 21 (13) unclassified	ORR	49 (41–57)	18 (14–NR) 1-year PFS = 63%	NR 1-year OS = 82%

ORR: overall response rate, mPFS: median progression-free survival, OS: overall survival, NA: not assessed, NR: not reached, NE: not evaluated.

The emerging clinical benefit of the vascular epidermal growth factor receptor (VEGFR) TKIs in the management of nccRCC prompted research examining the efficacy of other drugs within this class, such as pazopanib and the second-generation TKI axitinib. Axitinib has a higher affinity for VEGFR-1, VEGFR-2, and VEGFR-3, compared to sunitinib and pazopanib (21). In a cohort of predominantly pRCC (n = 22; 66% pRCC and 10% chRCC), pazopanib demonstrated promising therapeutic value, with an ORR of 28%, an mPFS of 16.5 months, and 1-year OS proportion of 69% (22). When stratified by histology, pRCC patients (n = 18) had an ORR of 39% and an mPFS of 17.3 months, whereas chRCC patients (n = 8) had an ORR of 33% and an mPFS of 18.3 months. In another phase II trial of nccRCC (n = 40; 60% pRCC and 10% chRCC), axitinib offered clinical benefit if used after treatment failure with temsirolimus. Park et al. reported an ORR of 37.5%, an mPFS of 7.4 months, and a median OS of 12.1 months (23). Although no trial has prospectively compared the efficacy of pazopanib and axitinib head-to-head, the similar ORR and mPFS data across trials highlight the utility of next-generation VEGFR TKIs in the treatment of nccRCC. Furthermore, the study conducted by Park et al. indicates that VEGFR TKIs could play a role in salvage therapy after failure with an mTOR inhibitor (23).

Given that single-agent therapies demonstrate only modest overall responses to nccRCC, two prospective phase II trials examined the efficacy of everolimus plus bevacizumab, a monoclonal antibody against VEGF-A. Voss et al. reported an mPFS of 11.0 months, an OS of 18.5 months, and an ORR of 29% (n = 34) (24); these outcomes are favorably, compared to monotherapy outcomes in ESPEN and ASPEN. Median PFS varied by histology; three out of five patients with chRCC achieved an mPFS > 6 months (and remained on treatment for >12 months), compared to two out of four patients with pRCC and no out of two patients with medullary RCC. Patients with medullary RCC achieved little or no benefit from everolimus plus bevacizumab. For patients with unclassified RCC (uRCC), the presence of a major papillary component correlated strongly with ORR (43% vs. 11%), mPFS (12.9 vs. 1.9 months), and median OS (28.2 vs. 9.3 months) (24). Although pathologic re-review determined that these patients did not meet sufficient criteria for a formal diagnosis of pRCC, the number of oncogenic variants within the spectrum of pRCC and uRCC with papillary morphology suggests that there may have a benefit for this combination within the genomic landscape of pRCC. Furthermore, a phase II trial (n = 37; 35% pRCC and 62% uRCC with papillary features) of everolimus plus bevacizumab reported an OS of 33.9 months, an mPFS of 13.7 months (6-month PFS of 78%), and an ORR of 35% (25). This validated the results of Voss et al., and strengthened everolimus plus bevacizumab as a robust therapeutic regimen for both pRCC and uRCC with papillary features subgroups (24).

Although VEGFR and mTOR inhibitors remain viable treatment options for nccRCC, the development of ICIs has revolutionized the treatment of solid tumors, and RCC is no exception. Data on their efficacy in clear cell RCC ushered in a new era of clinical trials focused on the treatment of nccRCC with ICIs. CheckMate 374 was the first trial to confirm the safety and efficacy of nivolumab, a PD-1 inhibitor, in a cohort of patients with nccRCC (n = 44; 55% pRCC and 15% chRCC). CheckMate 374 reported an ORR of 13.6%, an mPFS of 2.2 months, and an OS of 16.3 months (26). Clinically meaningful antitumor activity was observed in patients regardless of the baseline tumor PD-L1 expression as well as in patients with chromophobe and collecting duct histologies, which historically demonstrate poor prognoses.

McGregor et al. also reported meaningful clinical efficacy across several nccRCC subtypes, including CDC and RMC, using a combination regimen of atezolizumab, a PD-L1 inhibitor, plus bevacizumab, a VEGF inhibitor (27). In their phase II trial of nccRCC patients (n = 42; 29% pRCC and 24% chRCC), McGregor et al. reported an ORR of 26% and an mPFS of 8.3 months. ORR was not significantly different across patients who had received prior systemic therapy versus those who had not received such therapy (31% vs. 38%), but ORR significantly improved in PD-L1 positive patients (n = 9) versus PD-L1 negative patients (n = 4) (60% vs. 19%, P = 0.01). Moreover, there was no statistically significant difference in ORR associated with histology, although an ORR of 40% was reported for patients with collecting duct histology (n = 5) and 100% for patients with RMC histology (n = 1). Although the response proportions from the collecting duct and RMC patients are limited due to the small sample size, these results are notable, given the poor prognosis and low response proportions reported historically with these rare subtypes.

Immunotherapy in combination with cabozantinib, a third-generation TKI with mesenchymal epithelial transition (*MET*) gene inhibitory activity that may enhance response to ICIs, was assessed in two recent trials (28). COSMIC-021 investigated the combination of atezolizumab plus cabozantinib in 32 patients with nccRCC (47% pRCC and 25% chRCC), which demonstrated an ORR of 31% and an mPFS of 9.5 months (1-year progression-free survival [PFS] of 39%) (29). The ORR was higher among patients with pRCC than among patients with chRCC (47% vs. 11%). Responses were observed regardless of the PD-L1 status. In a trial of 40 patients with nccRCC (80% pRCC) taking nivolumab plus cabozantinib, Lee et al. reported an ORR of 47.5%, an mPFS of 12.5 months, and an OS of 28 months (30). Seven additional patients, all with chRCC histology, enrolled in the trial as cohort 2; the ORR in this trial was 0% with no partial response, and thus the cohort was closed early because of lack of efficacy. Taken together, these results suggest an improvement in outcomes for patients treated with ICI plus cabozantinib, compared to ICI alone (ORR of 31% and 47.5% vs. 13.6% in CheckMate 374). All three trials had similar proportions of patients who had received prior systemic therapy (22% vs. 35% vs. 34%). Although the efficacy of ICI therapy remains mixed with respect to the chRCC subtype, the COSMIC 021 trial highlights the therapeutic advantage of cabozantinib with respect to pRCC in particular. This is attributed to the action of cabozantinib, which targets receptor tyrosine kinases associated with the *MET* gene—mutations of which are commonly identified in patients with pRCC histology.

The promising therapeutic benefit of combination therapy continued to be explored with the CheckMate 920 trial. Given the clinical benefit of combination ICI observed in metastatic ccRCC setting, Checkmate 920 was designed to assess the role of nivolumab plus ipilimumab in nccRCC patient (31). This phase IIIb/IV trial studied 52 treatment-naïve nccRCC patients (42% uRCC, 35% pRCC, and 13% chRCC), who were given nivolumab plus ipilimumab, an ICI that inhibits CTLA-4. CheckMate 920 reported an ORR of 19.6%, an mPFS of 3.7 months, and an OS of 21.2 months (32). ORR improved in PD-L1 positive patients, compared to PD-L1 negative patients, but this difference was not statistically significant (30.8% vs. 14.3%, respectively). Of note, 2 patients achieved complete response (1 pRCC and 1 uRCC patient) and 7 patients achieved partial response (4 pRCC and 3 uRCC), while the remaining patients (n = 17) had a stable disease. The results of this study demonstrate encouraging antitumor activity and similar treatment responses for this regimen, compared to ICI–VEGF-targeted agent combinations.

In the largest prospective clinical trial of nccRCC patients to date, KEYNOTE-B61 examined the role of pembrolizumab plus lenvatinib, a multitargeted VEGF TKI, in 158 treatment-naïve patients (59% pRCC and 18% chRCC). KEYNOTE-B61 reported an ORR of 49%, an mPFS of 18 months, and a 12-month OS proportion of 82% (33). Consistent results were observed across histological subtypes and with presence or absence of sarcomatoid features. Responses were durable, with approximately 75% of responders remaining in response for at least 12 months. Given the excellent antitumor activity of pembrolizumab plus lenvatinib, KEYNOTE-B61 supports this combination as a first-line treatment option for patients with advanced nccRCC, regardless of histology.

### Papillary

Papillary renal cell carcinoma is the most common form of nccRCC, and it represents 10–15% of all RCCs (6). Previously, pRCC was split into type I and type II pRCC, but additional studies on the immunohistochemical spectrum of pRCC have reformed the clinical landscape of pRCC (34, 35). This prompted the World Health Organization (WHO) to eliminate the distinction between the type I and type II pRCC sub-categorization in the Classification of Tumors of the Urinary and Male Genital Systems 2022 (36). pRCC has been linked to specific genetic mutations, many of which are also associated with hereditary RCC syndromes. Notably, both sporadic and hereditary papillary renal carcinoma (HPRC) are tied to mutations of the *MET* oncogene on chromosome 7q31, leading to constitutive activation of this tyrosine kinase pathway and upregulation of cell proliferation signals (4, 37). The Cancer Genome Atlas (TCGA) study, which comprehensively characterized 161 pRCCs, found 81% of sporadic pRCCs harbor altered *MET* status (38), giving this kidney tumor subtype a selective growth advantage. HPRC patients often have an additional copy of chromosome 7 bearing the mutated allele, which is passed in an autosomal dominant manner, resulting in phenotypically bilateral multifocal papillary type I renal tumors (39).

Papillary renal cell carcinoma is also associated with Hereditary Leiomyomatosis and Renal Cell Carcinoma (HLRCC), although mutations in this syndrome have been localized to the *FH* gene on chromosome 1 (4, 37). In this autosomal dominant familial syndrome, inactivation of the *FH* tumor suppressor gene leads to the dysregulation of TCA cycle and the accumulation of HIF, resulting in the overexpression of proangiogenic growth factors, including VEGF and epidermal growth factor receptor (EGFR) (40). Identifying these specific molecular changes is important for classification within this histologically diverse group of malignancies and may ultimately inform clinicians the therapies that are likely to demonstrate the strongest efficacy. For example, two clinical trials are looking specifically at HLRCC-based therapies on the hypothesis that metabolic alterations secondary to *FH* inactivation would be susceptible to the VEGF inhibitor bevacizumab and the EGFR TKI erlotinib. Preliminary results from one of these trials reported an mPFS of 14.3 months and an ORR of 54.2%, with an ORR of 72% in the HLRCC cohort (n = 43) and an ORR of 35% in the sporadic pRCC cohort (n = 40) (NCT01130519) (41, 42). Because this is the first and largest prospective study of bevacizumab plus erlotinib in HLRCC, these drugs must be offered as the preferred treatment regimen in HLRCC patients. The second trial recruited HLRCC patients for treatment with bevacizumab plus erlotinib in combination with the PD-L1 inhibitor atezolizumab (NCT04981509) (43).

Since pRCC is the most prevalent histologic variant of nccRCC, several prospective trials have focused on treatment for this subtype of nccRCC (Table 2). In 2009, SWOG S0317 was the first prospective trial to specifically examine the pRCC subtype of nccRCC. This phase II trial looked at the efficacy of erlotinib, a TKI that acts on EGFR in 45 patients. SWOG S0317 reported an ORR of 11% with an OS of 27 months (44). Given these encouraging results with erlotinib in pRCC, the SWOG S1107 trial investigated the efficacy of erlotinib beside tivantinib (n = 25), a selective *MET* gene inhibitor, compared to tivantinib alone (n = 25). Compared to the tivantinib alone group, the tivantinib plus erlotinib group demonstrated modestly increased mPFS (3.9 months vs. 2.0 months) and OS (11.3 months vs. 10.3 months) (45). However, both groups had an ORR of 0%, and the study was closed due to a lack of clinical activity. One explanation for the lack of efficacy in SWOG S1107, compared to SWOG S0317, was that only one patient in SWOG S1107 harbored a *MET* mutation in the tyrosine kinase domain, underscoring the importance of tailoring nccRCC therapy based on the genomic and molecular profile of the tumor.

**Table 2:** Papillary histology.

Agent (trial, year)	Phase	N per group	Primary endpoint	ORR, % (95% CI)	mPFS, months (95% CI)	OS, months (95% CI)
Erlotinib (SWOG S0317, 2009)^(44)^	II	45	ORR	11 (3–24)	–	27 (13–36)
Tivantinib vs. tivantinib + erlotinib (SWOG S1107, 2017)^(45)^	II	25 Tivantinib 25 Tivantinib + erlotinib	ORR	0 in both arms (closed early)	2.0 (1.8–30) 3.9 (1.8–3.0)	10.3 (7.3–15.7) 11.3 (6.7–21.9)
Sunitinib (SUPAP, 2015)(^46)^	II	15 Type I 47 Type II	ORR	13 11	6.6 (2.8–14.8) 5.5 (3.8–7.1)	17.8 (5.7–26.1) 12.4 (8.2–16)
Foretinib (2013)^(47)^	II	74	ORR	13.5 (6.7–23.5)	9.3 (6.9–12.9)	NR 1-year OS: 70%
Savolitinib vs. sunitinib (SAVOIR, 2020)^(48)^	II	33 Savolitinib 27 Sunitinib	PFS	27 (13.3–45.5) 7 (0.9–24.3)	7.0 (2.8–NR) 5.6 (4.1–6.9) HR: 0.71 (0.37–1.36), p = 0.31	NR (11.9–NR) 13.2 (7.6–NR) HR: 0.51 (0.2–1.2), P = 0.11
Savolitinib (2017)^(49)^	II	44 *MET*(+) 46 *MET*(-) 19 *MET*(?)	ORR	18 0 –	6.2 (4.1–7.0) 1.4 (1.4–2.7) –	– – –
Crizotinib (CREATE, 2017)(^50)^	II	4 *MET*(+) 16 *MET*(-) 3 *MET*(?)	ORR	50 (6.8–93.2) 6.3 (0.2–30.2) 33.3 (0.8–90.6)	1-year PFS: 75.0% (12.8–96.1) 1-year PFS: 27.3% (8.5–50.4) 1-year PFS: 66.7% (5.4–94.5)	1-year OS: 75.0% (12.8–96.1) 1-year OS: 71.8% (41.1–88.4) 1-year OS: 100%
Sunitinib vs. cabozantinib vs. crizotinib vs. savolitinib (SWOG 1500/PAPMET, 2021)^(51)^	II	29 Savolitinib 28 Crizotinib 46 Sunitinib 44 Cabozantinib	PFS	3 0 4 23	3.0 (2.8–7.2) 2.8 (2.6–3.6) 5.6 mo (3–7) 9.0 (6–12) HR: 0.60 (0.37–0.97), P = 0.019	11.7 19.9 16.4 20.0 HR: 0.84 (0.47–1.51)
Everolimus (RAPTOR, 2016)^(53)^	II	88	PFS	1	4.1 (3.6–5.5)	21.4 (15.4–28.4)
Sunitinib vs. everolimus (ESPEN, 2016)^(18)^	II	13 Sunitinib 14 Everolimus	PFS	NA NA	5.7 (1.4–19.8) 4.1 (1.5–7.4)	16.6 (5.9–NA) 14.9 (7.1–22.7)
Sunitinib vs. everolimus (ASPEN, 2016)^(19)^	II	33 Sunitinib 37 Everolimus	PFS	NA NA	8.1 (5.8–11.1) 5.5 (4.4–5.6)	NA NA
Levantinib + everolimus (2021)^(54)^	II	20	ORR	15 (3–38)	9.2 (3.5–NE)	11.7 (8.1–NE)
Pembrolizumab (Keynote 427 Cohort B, 2021)(^55)^	II	118	ORR	28.8 (20.8–37.9)	5.5 (3.9–6.9)	31.5 (25.5–NR)
Pembrolizumab + lenvatinib (KEYNOTE-B61, 2023)^(33)^	II	93	ORR	54 (43–64)	NA	NA

ORR: overall response rate, mPFS: median progression-free survival, OS: overall survival, NA: not assessed, NR: not reached, NE: not evaluated.

The prospective phase II trial, SUPAP, reported promising findings in 62 pRCC patients treated with the VEGFR-inhibitor sunitinib; type I pRCC patients had an ORR of 13%, an mPFS of 6.6 months, and an OS of 18.7 months, and type II pRCC patients had an ORR of 11%, an mPFS of 5.5 months, and an OS of 12.4 months (46). A third phase II clinical trial, which looked at 74 pRCC patients treated with foretinib, a TKI with c-*MET* inhibitor activity, reported an ORR of 13.5%, an mPFS of 9.3 months, and a 1-year OS of 70% (47). Of note, the presence of a germline *MET* mutation in pRCC was highly predictive of response compared to the patients without germline *MET* mutations (50% response vs. 9% response). Although mPFS in the foretinib study improved relative to the mPFS reported by SWOG S0317 and SUPAP, ORR was clinically equivalent across trials of these three distinct agents (11% vs. 13% vs. 13.5%).

Increasing evidence on the implication of mutations in *MET* gene in pRCC prompted investigators to examine selective *MET* inhibitors, such as savolitinib. The SAVOIR trial randomized 60 pRCC patients to either savolitinib or sunitinib, reporting that ORR, mPFS, and OS were numerically greater in the savolitinib group, compared to the sunitinib group, yet none of these results were statistically significant (ORR 27% vs. 7%, mPFS 7.0 months vs. 5.6 months, and OS not reached (NR) vs. 13.2 months) (48). The SAVOIR trial was followed by a larger multicenter phase II trial examining savolitinib in pRCC patients stratified by *MET* status (n = 109). Choueiri et al. reported a significantly improved treatment response in the *MET*-positive group, compared to the *MET*-negative group, with an ORR of 18% versus 0% (49). Median PFS was also significantly improved in the *MET*-positive group, compared to the *MET*-negative group (6.2 months vs. 1.4 months). These encouraging results highlighted the functioning of savolitinib in the treatment of *MET*-driven pRCC and corroborated earlier findings with the c-MET inhibitor foretinib.

The CREATE trial examined crizotinib, a TKI with c-*MET* inhibitor activity, in 23 pRCC patients stratified by *MET* status. These investigators reported an ORR of 50% in the *MET*-positive cohort compared to an ORR of 6.3% in the *MET*-negative cohort. One-year PFS was 75.0% in *MET*-positive patients compared to 27.3% in *MET*-negative patients, but 1-year OS was similar between both groups (75.0% vs. 71.8%, respectively) (50). The ORR in CREATE reflects favorably on the ORRs demonstrated in SAVOIR and Choueiri et al., indicating that crizotinib could also serve as a viable treatment option for patients with advanced pRCC (49). A source of variability within these studies was related to how broadly these investigators defined *MET*-positive status. *MET*-positive status was considered any mutation within the gene, or it was more strictly defined as a mutation within the tyrosine kinase domain. Additionally, *MET*-driven disease can be secondary to a variety of abnormal activation mechanisms, such as gene amplification or chromosome 7 copy gain.

To directly compare several previously discussed agents in pRCC patients, PAPMET (SWOG 1500), randomized 147 patients to either savolitinib, crizotinib, sunitinib, or cabozantinib. PAPMET halted recruitment in savolitinib and crizotinib groups because of HR for mPFS > 1, compared to that of sunitinib at interim analysis. Final analysis of the PAPMET trial demonstrated an mPFS of 9.0 months for cabozantinib, 5.6 months for sunitinib, 3.0 months for savolitinib, and 2.8 months for crizotinib. The ORR was 23% with cabozantinib, 4% with sunitinib, 3% with savolitinib, and 0% with crizotinib. The OS was 20.0 months with cabozantinib, 16.4 months with sunitinib, 11.7 months with savolitinib, and 19.9 months with crizotinib; none of these differences in median OS were statistically significant (51).

The PAPMET trial was an important turning point in the treatment landscape of nccRCC because it was the first trial to show a clinically and statistically significant improvement in mPFS and ORR with the third-generation TKI, cabozantinib, over the existing standard of care, sunitinib. Moving forward, cabozantinib would become the standard of care for these patients. Furthermore, selective *MET* inhibitors, such as savolitinib and crizotinib, did not appear to have superior clinical activity compared to sunitinib, despite the modest improvement with savolitinib therapy observed in SAVOIR and the significant improvement with crizotinib observed in CREATE. However, PAPMET did not use a biomarker design, and the unknown *MET* status of the tumors in this study may explain why the savolitinib and crizotinib groups had inferior efficacy in this trial but not in others. To further investigate the promising clinical activity demonstrated by selective *MET* inhibitors, such as savolitinib and crizotinib, an ongoing phase II clinical trial (NCT02019693) is assessing the antitumor response of pRCC patients to capmatinib, a selective *MET* inhibitor currently approved for treating non-small cell lung cancer (52).

Everolimus as a single-agent therapy was first examined in the pRCC subgroup of the RAPTOR trial. RAPTOR examined 88 pRCC patients treated with everolimus, reported an ORR of 1%, an mPFS of 7.9 months for type I pRCC and 5.1 months for type II pRCC, and an OS of 28 months for type I pRCC and 24.2 months for type II pRCC (53). The ESPEN trial, which directly compared everolimus to sunitinib, reported an mPFS of 4.1 months and an OS of 14.9 months for pRCC patients receiving everolimus (n = 13), compared to an mPFS of 5.7 months and an OS of 16.6 months for patients receiving sunitinib (n = 14) (18). The ASPEN trial reported similar findings in its pRCC cohort; patients receiving everolimus showed an mPFS of 5.5 months (n = 37) compared to an mPFS of 8.1 months for patients receiving sunitinib (n = 33) (19). Results across treatment groups were not significant; thus, the ASPEN and ESPEN trials suggested that both mTOR inhibitors and TKIs are effective treatment strategies in pRCC patients. A 2021 phase II study of pRCC patients conducted by Hutson et al. examined the combination of everolimus plus lenvatinib, a multitargeted TKI, in 20 patients, reporting an ORR of 15%, an mPFS of 9.2 months, and an OS of 11.7 months (54). This suggested that the addition of a multitargeted TKI to mTOR monotherapy could improve ORR in pRCC patients.

The KEYNOTE-427 Cohort B trial is the largest prospective phase II clinical trial to date examining immunotherapy alone in nccRCC patients. This study examined 118 pRCC patients treated with pembrolizumab, a PD-1 inhibitor, and reported an ORR of 28.8%, an mPFS of 5.5 months, and an OS of 31.5 months for this cohort of patients. The ORR was three times higher in patients with a PD-L1 combined positive score >1, compared to patients with a PD-L1 combined positive score <1 (35.3% vs. 12.1%) (55). These results were favorable, compared to the outcomes reported in the above prospective clinical trials evaluating TKIs and mTOR inhibitors in pRCC patients. Furthermore, KEYNOTE-427 demonstrated similar outcomes reported in other ICI therapy trials such as CheckMate 374 (26) (ORR of 13.6%, mPFS of 2.2 months, and OS of 16.3 months for nccRCC patients, 55% pRCC, treated with nivolumab), CheckMate 920(32) (ORR of 19.6%, mPFS of 3.7 months, and OS of 21.2 months for 52 nccRCC patients, 35% pRCC, treated with nivolumab plus ipilimumab), and the Lee et al. study (30) (ORR of 47.5%, mPFS of 3.7 months, and OS of 21.2 months for nccRCC patients, 80% pRCC, treated with cabozantinib plus nivolumab). However, comparison of these trials is limited because the CheckMate trials and the Lee et al. study did not analyze outcomes for their pRCC subgroups specifically. Nevertheless, the large power of KEYNOTE-427 established pembrolizumab as a meaningful treatment option for patients with pRCC. That being said, the activity observed with pembrolizumab plus lenvatinib in KEYNOTE-B61 (33) (ORR of 54% for 93 pRCC patients) compared favorably with that of pembrolizumab alone observed in KEYNOTE-427, indicating that the addition of lenvatinib resulted in improved outcomes.

### Chromophobe

Chromophobe renal cell carcinoma is the second most common form of nccRCC, and it represents 4–5% of all RCCs (6). The TCGA study, which profiled 81 chRCC patients, found that 86% of chRCC patients demonstrate a pattern of chromosomal losses in chromosomes 1, 2, 6, 10, 13, and 17 (56). In particular, chRCC has been linked to Birt–Hogg–Dubé (BHD) syndrome, an autosomal-dominant disorder associated with germline mutations in the folliculin (*FLCN*) tumor suppressor gene on chromosome 17 (4, 37). In a review of 130 BHD patients, 34% of resected tumors had chRCC pathology, and an additional 50% of tumors were a hybrid of chromophobe and oncocytic histologies (57). Loss of *FLCN* leads to mTOR upregulation, adenosine monophosphate-activated protein kinase (AMPK)-driven mitochondrial biogenesis with increased production of *reactive oxygen species* (ROS), and dysregulation of lysosome dynamics (4, 58). In addition, chRCC has been linked to Cowden syndrome and alterations in the *PTEN* gene on chromosome 10q23; *PTEN*-deficient tumors also lead to mTOR upregulation through increased levels of phosphatidylinositol 3,4,5-trisphosphate (PIP3) (4).

Owing to the low incidence of chRCC, the availability of clinical trial data on outcomes stratified to patients with this subtype of nccRCC is limited (Table 3). Even in trials that analyzed outcomes within a chRCC subgroup, the results are further restricted by extremely small cohort sizes. In 2012, Tannir et al. reported an ORR of 40% and an mPFS of 12.7 months for treatment with sunitinib (n = 5) (59); the chRCC subgroup demonstrated a stronger antitumor response to sunitinib than the pRCC subgroup, which had an ORR of 0% and an mPFS of 1.6 months (n = 27). *KIT* gene overexpression is common in chRCC (60), so the durable response to therapy in chRCC, compared to pRCC, could reflect on the *KIT* inhibitory activity of sunitinib. In 2016, the ESPEN trial reported a similar mPFS of 8.9 months in chRCC subgroup of the sunitinib treatment arm of their trial. OS was 31.6 months for the sunitinib group (n = 6), compared to 25.1 months for the everolimus group (n = 6) (18). In their chRCC subgroup, the ASPEN trial reported an mPFS of 5.5 months for sunitinib (n = 10), compared to an mPFS of 11.4 months for everolimus (n = 6) (19). A lack of significance and a wide range of mPFS outcomes across treatment arms underscored that chRCC treatment response was unpredictable. Jung et al. reported an mPFS of 18.3 months with an OS of 18.9 months for treatment with pazopanib in chRCC (n = 3) (22), and Park et al. reported an mPFS of 11.0 months with an OS of 22.2 months for treatment with axitinib in chRCC (n = 4) (23). A few partial responses and no complete responses were observed across the various treatments in these trials, underscoring that chRCC remained highly variable in sensitivity to current therapeutic regimens (if not entirely unresponsive). For example, Lee et al. reported an ORR of 0% in their 2022 trial of cabozantinib plus nivolumab (n = 7) (30), even though this regimen showed promising efficacy in pRCC.

**Table 3:** Chromophobe histology.

Agent (trial, year)	Phase	N per group	Primary endpoint	ORR, % (95% CI)	mPFS, months (95% CI)	OS, months (95% CI)
Sunitinib (2012)^(59)^	II	5	ORR and PFS	40	12.7 (8.5–NA)	NA
Sunitinib vs. everolimus (ESPEN, 2016)^(18)^	II	6 Sunitinib 6 Everolimus	PFS	NA NA	8.9 (2.9–20.1) NA	31.6 (14.2–NA) 25.1 (4.7–NA)
Sunitinib vs. everolimus (ASPEN, 2016)^(19)^	II	10 Sunitinib 6 Everolimus	PFS	NA NA	5.5 (3.2–19.7) 11.4 (5.7–19.4)	NA NA
Pazopanib (2018)^(22)^	II	3	ORR	33	18.3 (11.9–24.7)	18.9
Axitinib (2018)^(23)^	II	4	PFS	NA	11.0	22.2
Cabozantinib + nivolumab (2022)^(30)^	II	7	ORR	0	NC	NC
Levantinib + everolimus (2021)^(54)^	II	9	ORR	44 (14–79)	13.1 (0.5–NE)	NE (0.5–NE)
Pembrolizumab (Keynote 427 Cohort B, 2021)^(55)^	II	21	ORR	9.5 (1.2–30.4)	3.9 (2.6–6.9)	23.5 (9.3–NR)
Pembrolizumab + lenvatinib (KEYNOTE-B61, 2023)^(33)^	II	29	ORR	28 (13–47)	NA	NA

ORR: overall response rate, mPFS: median progression- free survival, OS: overall survival, NA: not assessed, NR: not reached, NE: not evaluated.

The most robust data on systemic therapies for chRCC come from a recent study of everolimus plus Lenvatinib, the KEYNOTE 427 trial of pembrolizumab, and the KEYNOTE-B61 trial of pembrolizumab plus lenvatinib. Hutson et al. reported an ORR of 44% and an mPFS of 13.1 months for patients with chRCC treated with everolimus plus lanvatinib (n = 9) (54), which compared favorably to ORRs for chRCC patients observed with pazopanib in Jung et al. (22) (33%, n = 3), sunitinib in Tannir et al. (59) (40%, n = 5), and in the ASPEN trial (19) (10%, n = 10). Furthermore, in the KEYNOTE 427 trial, ORR was 9.5%, mPFS was 3.9 months, and OS was 23.5 months for the chRCC subgroup (n = 21); the chRCC subgroup responded poorly to immunotherapy, compared to the pRCC subgroup (55). In the KEYNOTE-B61 trial, ORR was 28% for the chRCC subgroup (n = 29); again, the chRCC subgroup responded poorly relative to the pRCC subgroup (33).The enhanced anticancer activity of lenvatinib plus everolimus and lenvatinib plus pembrolizumab relative to sunitinib and pembrolizumab alone in the chRCC subgroup supports the hypothesis that dual inhibition of the VEGFR and mTOR pathways is a preferred therapeutic strategy for this histology. mTOR inhibitors, such as everolimus, are preferred in patients with *FLCN* mutations, because the loss of *FLCN* leads to mTOR upregulation. However, the exact mechanism for poorer response to pembrolizumab in chRCC patients, compared to pRCC patients in KEYNOTE 427 and KEYNOTE-B61, remains unclear. Thus, consensus on optimal therapy for patients with chRCC remains complicated by substantial heterogeneity and humble efficacy across treatments as well as the rarity of this subtype, limiting the power of current research findings.

### Collecting Duct, Renal Medullary, Translocation, and Other nccRCC Subtypes

The remaining nccRCC subtypes are extremely rare, accounting for less than 1% of all nccRCC patients. CDC and RMC are associated with mutations in *SMARCB1* tumor suppressor gene, leading to defects in the chromatin-remodeling complex (61, 62). These forms of nccRCC represent orphan diseases that are often excluded from large, randomized prospective trials because of their paucity and dismal prognoses. Although clinically and pathologically distinct, CDC and RMC have been reported to behave more like aggressive urothelial carcinomas than RCCs, suggesting that these nccRCC subtypes may respond to platinum-based chemotherapy regimens (63–65).

Data for the effective treatment of RMC, which primarily affects younger patients with sickle cell trait and is refractory to antiangiogenic therapy (66), are limited to observational studies. Several case studies have lent evidence to the combination of gemcitabine and cisplatin in improving survival of patients with this highly lethal malignancy (67–69). In a retrospective analysis of 45 patients with RMC across eight academic institutions in North America and France who received platinum-based chemotherapy, the ORR was 29% and the OS for all patients was 13.0 months; only seven patients (<20%) survived for >24 months (70). Investigators are now studying treatment options for RMC patients who are resistant to platinum-based therapy because no effective salvage regimens have been established to date. In a retrospective analysis of 16 RMC patients who did not respond to platinum-based therapy, treatment with gemcitabine plus doxorubicin resulted in an mPFS of 2.8 months and a median OS of 8.1 months from initiation of gemcitabine plus doxorubicin (71). In another retrospective analysis of 10 platinum-refractory RMC patients, treatment with bevacizumab plus erlotinib resulted in an mPFS of 3.5 and a median OS of 7.3 months from bevacizumab plus erlotinibinitiation (72). Together, these studies suggest that gemcitabine plus doxorubicin or bevacizumab + erlotinib is clinically active as a salvage therapy in RMC.

Data for the effective treatment of CDC are also mostly observational. In a woman with CDC treated with three cycles of paclitaxel + carboplatin, Gollob et al. reported an 80% reduction in tumor burden (73), with complete regression of lymph node metastases and a significant shrinkage of renal mass. This patient was rendered free of disease through nephrectomy and was without recurrence during a 20-month follow-up. Peyromaure et al. reported an objective response to three cycles of gemcitabine plus cisplatin in two patients with T3N+M+ CDC (74); these patients remained disease-free at 27 and 9 months after nephrectomy. The exciting results from these case studies were validated in a prospective multicenter phase II study of 23 CDC patients treated with gemcitabine plus cisplatin or carboplatin (Table 4). Oudard et al. reported an ORR of 26%, an mPFS of 7.1, and an OS of 10.5 months (75).

**Table 4:** Renal medullary and collecting duct histology.

Agent (trial, year)	Phase	N per group	Primary endpoint	ORR, % (95% CI)	mPFS, months (95% CI)	OS, months (95% CI)
Gemcitabine + cisplatin or carboplatin (2007)^(75)^	II	23 Collecting ducts	ORR	26 (8–44)	7.1 (3–11.3)	10.5 (3.8–71.1)
Cabozantinib (BONSAI, 2022)^(78)^	II	23 Collecting ducts	ORR	35 (16–57)	4 (3–13)	7 (3–31)

ORR: overall response rate, mPFS: median progression-free survival, OS: overall survival.

Moreover, limited current data regarding ICI therapy for RMC and CDC are available (76), so these agents remain investigational. Thus, the European Society for Medical Oncology (ESMO) guidelines recommend platinum-based chemotherapy as the first-line treatment for these histologic subtypes (77). However, a recent prospective trial by Procopio et al., which evaluated 23 CDC patients treated with cabozantinib, reported an ORR of 35%, an mPFS of 4 months, and an OS of 7 months (78). These authors reported fewer grade 3 or higher adverse events with cabozantinib, compared to the gem/cis regimen administered in Oudard et al. (75). Consequently, cabozantinib emerged as an encouraging therapeutic option for patients with CDC.

Translocation RCC (tRCC) represents the subset of sporadic RCC driven by genetic rearrangements of microphthalmia-associated transcription factor (MiTF) family members, such as *TFE3* gene on chromosome Xp11 and *TFEB* on chromosome 6p21. In a review of 212 pediatric RCC cases, MiTF-tRCC was the primary histologic subtype in children, comprising 42% of all cases (79). MiTF fusion isoforms drive constitutive nuclear localization of MiTF proteins and stimulate the transcription of HIF (4), promoting carcinogenesis. Individuals carrying a germline mutation in MiTF were shown to have a five-fold increased risk of developing RCC (80). Prospective clinical trial data are again limited for this histology of nccRCC (Table 5). The ESPEN trial reported an mPFS of 6.1 and an OS of 16.2 for its tRCC cohort treated with sunitinib (n = 3) as well as an mPFS of 3.0 and an OS of 8.1 for its tRCC cohort treated with everolimus (n = 4) (18). These results were not significant compared to the responses observed in the pRCC and chRCC groups. However, Park et al. reported an mPFS of 11.1 and an OS of 16.9 for tRCC patients treated with axitinib after failed treatment with temsirolimus (n = 7) (23), which was accentuated, compared to the mPFS and OS of the entire nccRCC cohort (mPFS = 7.4 and OS = 12.1) as well as the individual pRCC and chRCC subgroups. Although the results are limited by the small number of patients analyzed, axitinib may have beneficial activity in the MiTF-translocation population. Evidence on the efficacy of VEGFR TKIs and mTOR inhibitors in this histologic subtype comes from retrospective studies. In a systematic review of 53 patients with *Xp11* tRCC, 33% of the patients showed an objective response to VEGFR-targeted and/or mTOR inhibitor treatment. The mPFS for patients treated with first-line sunitinib was 8.2 months (n = 11). Patients receiving second-, third-, or fourth-line treatment with sunitinib or sorafenib had an mPFS of 11 months (n = 3) and 6 months (n = 8), respectively. All patients who progressed on VEGFR-targeted therapy and were switched over to an mTOR inhibitor achieved stable disease (81). These results, while limited by their observational nature, report similar outcomes as the ones observed in prospective trials, and indicate that there may be a clinical benefit to switching to mTOR inhibitors after failure with VEFGR-targeted therapies in tRCC.

**Table 5:** MiTF-translocation histology.

Agent (trial, year)	Phase	N per group	Primary endpoint	ORR, % (95% CI)	mPFS, months (95% CI)	OS, months (95% CI)
Sunitinib vs. everolimus (ESPEN, 2016)^(18)^	II	3 Sunitinib 4 Everolimus	PFS	NA NA	6.1 (6.0–8.8) 3.0 (1.3–NA)	16.2 (8.8–NA) 8.1 (5.5–23)
Axitinib (2018)^(23)^	II	7	PFS	NA	11.1 (7.6–14.6)	16.9

ORR: overall response rate, mPFS: median progression-free survival, OS: overall survival, NA: not assessed.

The benefit of cabozantinib treatment reported by the COSMIC 021 and PAPMET trials in pRCC patients led investigators to examine the efficacy of this drug in tRCC patients, especially because tRCC is similar to pRCC as it harbors high expression of c-*MET*, and *TFE3* fusion proteins have been shown to bind to the *MET* promoter (82). In the largest retrospective study of cabozantinib treatment to date, Thouvenin et al. reported an ORR of 17.3%, an mPFS of 6.8 months, and an OS of 18.3 months for 52 patients with tRCC (88.5% *TFE3*) (83). Stable disease was the best response for 26 patients (50%), lasting for more than 6 months for 15 patients (29%), and progression was the best response for 17 patients (32.7%). Another large, multicenter, retrospective study of nccRCC patients treated with cabozantinib reported an ORR of 29% and a median time to treatment failure of 8.3 months in their tRCC subgroup (n = 17) (84). These studies demonstrated considerable and consistent antitumor efficacy for cabozantinib in tRCC patients, particularly those with *TFE3* gene translocations. However, both these studies are limited by their observational methodology as well as confounding from pretreatment with other drug classes; 78.8% of the patients in the study conducted by Thouvenin et al. had already been treated with VEGF TKI monotherapy, ICI therapy, or a combination (83). Thus, although these retrospective outcomes are promising, prospective clinical trial data are needed to establish the efficacy of cabozantinib in tRCC.

### Ongoing Clinical Trials

The results of the above-mentioned trials are undoubtedly advancing the landscape of systemic therapies for patients with advanced or metastatic nccRCC. Despite recent advances in the treatment of this disease, there is still a dire need to improve the bleak outcomes for these patients. Several clinical trials are currently underway to examine combination treatment regimens as well as novel therapeutic options for advanced or metastatic nccRCC (Table 6).

**Table 6:** Ongoing clinical trials.

Clinical trial	Phase	Experimental and control arms	Histology	Outcomes (*primary)	Estimated primary completion date
NCT02019693^(52)^	II	Capmatinib (INC280)	RCC	ORR at 4–5 years*	December 2021 (no results posted yet)
NCT04535687^(93)^	II	Fluzoparib	RCC	ORR at 3 months* Radiographic PFS at 6 months OS at 18 months TTP at 6 months Adverse events	March 2022 (no results posted yet)
NCT04644432^(95)^ (INDIGO)	II	For patients with a DNA mutation that matches a targeted treatment, one of the following are administered: erlotinib, osimertinib, alectinib, dabrafenib + trametinib, trastuzumab, olaparib, pembrolizumab, cabozantinib, crizotinib, palbociclib, imatinib vs. sunitinib for patients with an angiogen profile vs. nivolumab for patients with an immune profile (or no immune, angiogen, or targeted mutation profile)	RCC and other urogenital neoplasms	ORR at 30 months* TTF at 30 months* OS at 30 months PFS at 30 months Disease control rate at 30 months Patient-reported outcomes via PRO-CTCAE questionnaire QoL per EORTC QLQ-C30 questionnaire Adverse events	September 2022 (no results posted yet)
NCT03685448^(92)^ (ANZUP)	II	Cabozantinib	RCC, pRCC, chRCC, tRCC, RCC with sarcomatoid components	ORR at 2 years* Adverse events PFS at 2 years OS at 2 years	October 2022 (no results posted yet)
NCT01130519^(42)^	II	Bevacizumab + erlotinib	HLRCC, sporadic pRCC	ORR at 4–5 years^*^ PFS Duration of response OS at 4–5 years	December 2022 (no results posted yet)
NCT 03866382^(91)^ (ICONIC)	II	Cabozantinib + nivolumab + ipilimumab	pRCC, chRCC, CDC, RMC, RCC with sarcomatoid components, and other rare GU tract cancers (bladder, prostate, testicular, penile)	ORR up to 5 years* Duration of response (CR or PR up to 5 years) PFS up to 5 years Overall survival up to 5 years Clinical benefit rate up to 5 years Incidence of adverse events Effects of treatment in patients with bone-only disease	February 2023 (no results posted yet)
NCT02819596^(94)^ (CALYPSO)	Ib	Savolitinib vs. durvalumab vs. savolitinib + durvalumab vs. tremelimumab + durvalumab	ccRCC and pRCC	Phase Ib: dose limiting toxicity^*^ ORR at 18 months in clear cell RCC^*^ ORR at 18 months in PRCC^*^ Pharmacokinetics PFS at 18 months OS at 18 months	March 2023 (no results posted yet)
NCT03117309^(86)^ (HCRN: GU16-260)	II	Nivolumab vs. nivolumab + ipilimumab	RCC	PFS at 1 year^*^ ORR at 1 year RR at 1 year CR, PR, and SD at 1 year PFS at 1 year Toxicity	September 2023
NCT04090710^(89)^ (CYTOSHRINK)	II	Ipilimumab + nivolumab followed by SBRT vs. ipilimumab + nivolumab	RCC	PFS at 2 years^*^ Safety (incidence of death) OS at 2 years ORR at 1 year QoL per EORTC QLQ-C30 questionnaire Adverse events Drug tolerability and discontinuation rates Changes in blood immune signatures and changes in stool microbiome	December 2023
NCT04413123^(90)^	II	Cabozantinib + nivolumab + ipilimumab	RCC and nccRCC (pRCC, chRCC, tRCC, CDC, RMC), unclassified RCC	ORR up to 21 months^*^ Duration of response PFS up to 21 months OS Adverse events QoL per FKSI-19 scale and BFI questionnaire	December 2024
NCT04981509^(43)^	II	Bevacizumab + Atezolizumab + Erlotinib	HLRCC, sporadic pRCC	Incidence of adverse events up to 28 days after treatment^*^ ORR up to 3 years (CR + PR)^*^ Disease control rate up to 3 years^*^ PFS up to 3 years^*^ OS up to 3 years^*^ Duration of response up to 3 years^*^ Response to treatment up to 3 years^*^	December 2024
NCT03075423^(85)^ (SUNNIFORECAST)	II	Ipilimumab + nivolumab vs. standard of care decided by physician	nccRCC (at least 50% non-clear cell component according to WHO classification)	OS at 12 months^*^ OS at 6 and 18 months OS at 5 years PFS at 5 years ORR at 4 years Incidence of TRAE QoL per FKSI–DRS questionnaire	June 2025
NCT04267120^(87)^ (LENKYN)	II	Lenvatinib + pembrolizumab	RCC	ORR at 2 years^*^ Safety and tolerability measured via adverse events PFS at 3 months and at 12 months OS at 6, 12 , 18 months, and up to 4 years	July 2027

ORR: overall response rate, PFS: progression-free survival, OS: overall survival, TTP: time to progression, QoL: quality of life, RR: response rate, CR: complete response, PR: partial response, SD: stable disease.

Immunotherapy as a preferred treatment for nccRCC has been investigated in the SUNNIFORECAST trial (NCT03075423), which is testing nivolumab and ipilimumab against sunitinib (85). The HCRN-GU16-260 trial (NCT03117309) investigates nivolumab alone versus nivolumab plus ipilimumab in advanced RCC (86). No optimal ICI therapy combination is clear yet, and therefore the LENKYN trial (NCT04267120) recruits treatment-naïve nccRCC patients for treatment with pembrolizumab plus lenvatinib (87). Preliminary results from the NEMESIA study, a subgroup analysis of the I-RARE (Meet-URO 23) trial, which were presented at the ESMO Congress 2022, showed that treatment with pembrolizumab plus axitinib resulted in an 86% disease control in patients with pRCC and chRCC (n = 25) (88). The CYTOSHRINK trial (NCT04090710) further evaluates the efficacy of immunotherapy by studying nccRCC patients ineligible for cytoreductive nephrectomy—the trial investigates stereotactic body radiation therapy in addition to ipilimumab plus nivolumab versus ipilimumab plus nivolumab alone (89).

Building on evidence from earlier trials, cabozantinib is currently being studied in combination with both nivolumab and ipilimumab (NCT04413123) (90). Additionally, the ICONIC trial (NCT03866382) evaluates this triple therapy regimen across a broader range of genitourinary cancers, including bladder, prostate, testicular, and penile cancers, in addition to nccRCC (91). The ANZUP trial (NCT03685448) evaluates cabozantinib in patients that are either unsuitable or have previously failed treatment with immunotherapy (92).

Given the modest efficacy of the existing treatments, several clinical trials also examines novel therapeutic regimens and drugs that have yet to be approved by US Food and Drug Administration (FDA) for treating RCC. One trial (NCT04535687) recruits patients for treatment with fluzoparib, an inhibitor of poly-ADP-ribose polymerase (PARP) types 1 and 2, which is also being studied in clinical trials as a treatment for advanced ovarian cancer (93). The CALYPSO trial (NCT02819596) compares whether savolitinib alone, durvalumab alone, savolitinib plus durvalumab, or tremelimumab plus durvalumab can demonstrate an antitumor effect in pRCC (94). Durvalumab, a PD-L1 inhibitor, has been approved for urothelial, lung, and biliary cancers, while tremelimumab, an anti-CDLA-4 monoclonal antibody used in phase II clinical trials for melanoma and mesothelioma, has yet to be approved by FDA.

The INDIGO trial (NCT04644432) plans to test first-line individualized treatment strategies based on DNA and RNA analyses of the tumors in nccRCC patients. Patients are allocated into four treatment arms containing 14 different treatment options (95). The INDIGO trial is especially exciting because it is the first study to assess outcomes using personalized medicine for rare RCC types—an essential next step in the treatment of a heterogenous malignancy with limited high-quality outcome data and no consensus on preferred treatment. Preliminary results from all these trials are eagerly awaited.

## Discussion and the Future Perspective

This review surveyed the existing prospective clinical trial data for treating nccRCC. Presently, therapeutic strategies are based mostly on phase II trials with small sample sizes, reflecting a scarce level of evidence to manage this disease. It is for this reason, subset analyses looking at differences in baseline characteristics, such as differences in response between males and females, are not currently feasible.

In trials that examined outcomes for a grouped histology nccRCC cohort, ORR ranged from 28% to 37.5% for treatment with VEGFR TKIs, 29–35% for treatment with mTOR inhibitors, and 13.6–49% for treatment with ICI-based therapies. Median PFS ranged from 6.1 to 16.5 months for treatment with VEGFR TKIs, 4.1–13.7 months for treatment with mTOR inhibitors, and 2.2–18 months for treatment with ICI-based therapies. Median OS ranged from 12.1 to 19.8 months for treatment with VEGFR TKIs, 14.9–33.9 months for treatment with mTOR inhibitors, and 16.3–28 months for treatment with ICI-based therapies. Of note, the ESPEN, ASPEN, and CESAR trials demonstrated that the TKI sunitinib offers greater efficacy over the mTOR inhibitor everolimus for nccRCC (18, 19). Checkmate 374 and Checkmate 920 also demonstrated clinically meaningful antitumor activity in nccRCC patients treated with immunotherapies such as nivolumab and nivolumab plus ipilimumab. Lastly, the KEYNOTE-B61 trial demonstrated that combination therapy with pembrolizumab plus lenvatinib has extremely promising antitumor activity across all nccRCC histologic subtypes and should be considered as first-line therapy.

In trials that specifically examined the pRCC histology, ORR ranged from 0% to 39% for treatment with VEGFR TKIs (0–50% if *MET*-selective), 1–15% for treatment with mTOR inhibitors, and 28.8–54% for treatment with ICI-based therapies. Median PFS ranged from 1.6 to 17.3 months for treatment with VEGFR TKIs (2.0–9.3 if *MET*-selective), 5.1–9.2 months for treatment with mTOR inhibitors, and 5.5 months for treatment with ICI-based therapies. Median OS ranged from 11.3 to 27 months for treatment with VEGFR TKIs (10.3–20 if *MET*-selective), 11.7–28 months for treatment with mTOR inhibitors, and 31.5 months for treatment with ICI-based therapies. The COSMIC 021 trial (29) and the Lee et al. study (30) demonstrated efficacy for the therapeutic combination of ICI plus cabozantinib, especially in the pRCC subgroup because of *MET* inhibitor advantage of cabozantinib. The Choueiri et al. study (49) and CREATE trial (50) demonstrated that savolitinib and crizotinib are also efficacious for treating *MET*-driven pRCC. Moreover, Voss et al. and Feldman et al. demonstrated that everolimus plus bevacizumab is particularly efficacious in patients with pRCC histology (24, 25). Lastly, preliminary results from the ongoing clinical trial (NCT01130519) suggest bevacizumab plus erlotinib as the preferred treatment option in *FH*-deficient HLRCC patients (41, 42).

In trials that specifically examined the chRCC histology, ORR ranged from 10% to 40% for treatment with VEGFR TKIs, 44% for treatment with mTOR inhibitors, and 0–28% for treatment with ICI-based therapies. Median PFS ranged from 5.5 to 18.3 months for treatment with VEGFR TKIs, 11.4–13.1 months for treatment with mTOR inhibitors, and 3.9 months for treatment with ICI-based therapies. Median OS ranged from 18.9 to 31.6 months for treatment with VEGFR TKIs and 23.5 months for treatment with ICI-based therapies; no reported median OS values were available across trials for treatment with mTOR inhibitors in the chRCC group. The KEYNOTE 427 trial demonstrated that pembrolizumab is active in chRCC (55), while Hutson et al. and KEYNOTE-B61 demonstrated that lenvatinib plus everolimus or lenvatinib plus pembrolizumab could be a preferred therapeutic strategy for chRCC relative to both sunitinib and immunotherapy alone (33, 54).

Very few prospective clinical trials have examined treatment outcomes for the CDC, RMC, and tRCC histologies. Presently, platinum-based chemotherapy remains the recommended treatment strategy for CDC and RMC (96), although Procopio et al. demonstrated that cabozantinib could be an efficacious option for CDC (78). Park et al. demonstrated that axitinib offers efficacy as a salvage therapy in nccRCC, especially the tRCC subgroup, after treatment failure with an mTOR inhibitor (23).

It is important to note that while sarcomatoid differentiation is not a true histologic classification because it can occur in any RCC histology at variable proportions, sarcomatoid features are correlated with a positive response to immunotherapy, regardless of the formally diagnosed tumor histology (13, 27, 97). Furthermore, because clinical outcomes for ICI-based therapies are superior for this group, compared to treatment with sunitinib (98), ICIs should be prioritized as first-line treatment for these patients.

It is critical to consider the mechanism of action of ICI and its implication on its immune-related toxicities. The disinhibition of T-cell function can result in a wide spectrum of immune-mediated inflammatory adverse events, ranging from life-threatening colitis, hepatitis, and hypophysitis to maculopapular eruptions (99). In the solid tumor literature, the incidence of any grade immune-related adverse event has been reported at 72% for anti-CTLA-4 monotherapy and 66% with anti-PD-1/PD-L1 therapy, with an increasing incidence with combination regimens (100, 101). The growing application of ICI for managing solid organ tumors, including nccRCC, warrants the recognition and management of the adverse events that accompany it.

Overall, TKI, mTOR, and ICI therapies for nccRCC elicited variable yet poor response proportions and disease control for nccRCC across all included studies. Collective data from these prospective trials demonstrate that ORRs are unlikely to be greater than 50%, mPFS is unlikely to be more than 18 months, and median OS is unlikely to be more than 3 years, regardless of histologic subtype and therapy. Hence, the current NCCN guidelines for managing advanced or metastatic nccRCC recommend enrollment in clinical trials (16). For patients who do not wish to participate in clinical trials, VEGF inhibitors, such as sunitinib, axitinib, pazopanib, lenvatinib, savolitinib, crizotinib, cabozantinib, and bevacizumab, as well as mTOR inhibitors, such as everolimus and temsirolimus, are viable agents. Additionally, ICIs, such as nivolumab, pembrolizumab, atezolizumab, and ipilimumab, are promising treatment options, with new data suggesting that pembrolizumab + lenvatinib has a powerful antitumor efficacy. Ongoing clinical trials are hoping to answer outstanding questions about the efficacy and safety of these pharmaceutical agents in various TKI-m–TOR, TKI–ICI, and ICI–ICI combinations, with an emphasis on the *MET* inhibitor cabozantinib.

Given that metastatic nccRCC is a notoriously challenging malignancy to treat even with clinicians’ existing arsenal of systemic therapies, cytoreductive nephrectomy may also offer survival benefit to these patients. In a recent study of 100 nccRCC patients subdivided by histology, who underwent cytoreductive nephrectomy at Memorial Sloan Kettering Cancer Center between 1989 and 2018, estimated 2- and 5-year survival was 40.1% and 12.2%, respectively, with a median OS of 13.7 months. The presence of sarcomatoid features conferred worse overall survival whereas the presence of papillary features was a favorable prognostic feature (102). In a retrospective analysis of 851 nccRCC patients within the Surveillance, Epidemiology, and End Results (SEER) registry from 2001 to 2014, the cumulative incidence of 2-year cancer-specific mortality was 52.6% in patients who underwent cytoreductive nephrectomy, compared to 77.7% in patients did not have cytoreductive nephrectomy. Cancer-specific mortality after nephrectomy was lower in all histologic subtypes of nccRCC. In fact, incremental survival benefit analyses in patients with cancer-specific mortality-free survival ≤24 months yielded both statistically and clinically meaningful survival benefit of 3 months in patients who underwent cytoreductive nephrectomy (103). These findings were supported by a larger analysis of 1573 metastatic nccRCC patients in the SEER database from 2006 to 2015 that examined the effect of systemic therapy with or without cytoreductive nephrectomy on overall mortality. Although the proportion of cytoreductive nephrectomy decreased by 6.3%, while the rate of systemic therapy increased by 7.5%, the combination of cytoreductive nephrectomy and systemic therapy resulted in the lowest overall mortality, relative to cytoreductive nephrectomy alone or systemic therapy alone across all histological subtypes (104). However, the study was limited because it was unable to report data on patient comorbidities and performance status, and information on the type, duration, and treatment adherence to systemic therapy was not obtainable. Since the medical management of advanced nccRCC has rapidly evolved and the only evidence about surgical treatment is retrospective, the impact of cytoreductive nephrectomy is promising but difficult to ascertain relative to or in combination with systemic therapy.

Although nccRCC has been analyzed as a single pathologic entity in traditional clinical trials, modern research is examining patients based on specific nccRCC histology, rather than as an all-inclusive group (96). Therapeutic approaches based on distinct molecular alterations associated with each nccRCC subtype could significantly improve patient outcomes, especially in patients with hereditary syndromes (37). Similarly to ccRCC, precision medicine is becoming increasingly important for managing nccRCC (105). The Cancer Genome Atlas has comprehensively characterized various subtype-specific molecular markers (56), and results from prospective clinical trials have confirmed indications for tailoring treatment decisions in patients whose tumors have been genetically profiled. HPRC patients and the majority of sporadic pRCC patients harbor activating *MET* mutations (4), and therefore they should be treated with *MET*-specific inhibitors, such as cabozantinib. HLRCC syndrome is secondary to *FH* inactivation, which results in overexpression of VEGF and EGFR (37); these patients should be treated with a combination of bevacizumab and erlotinib. Even in sporadic nccRCC patients, tailoring therapy based on the genomic and metabolic profile of the tumor, such as cabozantinib for *MET*-driven pRCC or immunotherapy for tumors with high PD-L1 positivity, should be leveraged.

In light of the correlation between distinct molecular markers and treatment responsiveness, a North American multidisciplinary panel of 33 urologists, medical oncologists, and clinical geneticists reached 97% clinical consensus that certain renal tumor histologies, such as succinate dehydrogenase (SDH)-deficient, *FH*-deficient, and hybrid oncocytic tumors, should always lead to genetic risk assessment (106). The panel specifically recommended genetic testing for individuals with RCC who have the first- or second-degree same-lineage relatives. Moreover, patients with multifocal or bilateral disease should be tested due to the increased frequency of hereditary syndromes associated with germline mutations; timely identification of a hereditary syndrome, such as VHL disease, BHD, and HLRCC, is critical as this diagnosis can significantly influence operative and inoperative management (107). Multigene panel testing is the agreed-upon approach for suspected hereditary RCC in the absence of classic syndromic features, although these tests may identify mutations associated with unrelated conditions. Thus, if a specific syndrome is suspected, only a single gene test should be pursued (106). In line with the recommendations of the American Society of Clinical Oncology (ASCO), the panel agreed with 92% consensus that urologists and oncologists with expertise in these syndromes can offer pretest counseling in patients with suspected hereditary RCC, especially given the shortage of and limitations in accessing genetic counselors (106). Consensus statements of the panel were also concordant with the most recent NCCN guidelines, which state that patients with kidney cancer, aged <46 years, have bilateral or multifocal renal masses, and/or have at least one first- or second-degree relative with RCC should undergo genetic risk assessment (106).

The role of host immune status has been studied in the setting of ccRCC. Clear cell RCC has been historically considered an immunogenic tumor, and as such, multiple host factors are thought to contribute to both carcinogenesis and response to treatment (108). However, to our knowledge, the role of host immune status has not been extensively studied, specifically in the non-clear cell setting. Insights into such may contribute to improved therapeutic success in specific histological subtypes of nccRCC. Furthermore, the role of tumor flare as a mechanism of drug resistance to anti-VEGF therapy has been described in the metastatic RCC population. It is hypothesized that discontinuation of anti-VEGF therapy can accelerate both tumor growth and metastases, a phenomenon that has been labeled “tumor flare” in the literature (109). A retrospective study of 63 patients, of which 16% were nccRCC, who received either sunitinib (89%) or pazopanib (11%), was performed to evaluate the prognostic role of discontinuing anti-VEGF therapy on further growth of the tumor. In patients with pRCC, discontinuation of therapy was 100% attributable to disease progression with a growth rate higher than the cumulative data after discontinuation of anti-VEGF therapy (0.7 cm/month vs. 1.1 cm/month) (98). The future studies are required to assess the role of host immune status and tumor flare, specifically in the nccRCC population.

## Conclusion

Overall, nccRCC remains a heterogenous and difficult-to-treat disease with limited prospective clinical trial data to establish a preferred treatment regimen within nccRCC histologic subgroups or in this patient population as a whole. Patients diagnosed with advanced or metastatic nccRCC should always be counseled by their physician to participate in an ongoing clinical trial. Genomic and metabolic studies continue to provide insight on the molecular biology behind nccRCC, and this research is important for developing targeted treatments for nccRCC. Ultimately, continued research is required to further illuminate treatment strategies and to improve lackluster patient outcomes for this diverse malignancy.
